# Massive Recurrent Chylous Ascites Following Laparoscopic Inguinal Hernia Mesh Repair

**DOI:** 10.7759/cureus.101306

**Published:** 2026-01-11

**Authors:** Jack Abadi Chiriti, Roberto Castañeda Gaxiola, Martin Rodriguez Alvarado, Manuel E Marquina Ramírez, Edmundo Arias Gomez, Brianda P Gomez Olvera

**Affiliations:** 1 General Surgery, American British Cowdray Medical Center, Mexico City, MEX; 2 Vascular Surgery, American British Cowdray Medical Center, Mexico City, MEX; 3 Medicine, Instituto Politecnico Nacional, Mexico City, MEX

**Keywords:** chylous ascites, inguinal hernia, laparoscopic hernia repair, lymphatic leakage, surgical complications

## Abstract

Chylous ascites is a rare condition characterized by the accumulation of lipid-rich lymph within the peritoneal cavity. While typically secondary to malignancy or trauma, its occurrence following laparoscopic inguinal hernia repair is exceptional. While clinical findings are usually sufficient to diagnose an inguinal hernia and justify surgical intervention, this case demonstrates that rare lymphatic malformations can closely mimic common pathology. A high index of clinical suspicion and further imaging should be employed when diagnostic ambiguity exists to avoid such devastating iatrogenic complications. This case contributes a novel presentation and management approach to the literature.

A 35-year-old female, initially misdiagnosed with a right inguinal hernia, underwent elective laparoscopic transabdominal preperitoneal (TAPP) repair with mesh placement. Three weeks postoperatively, she developed progressive abdominal distension and weight loss. CT imaging revealed massive free fluid consistent with ascites, and paracentesis yielded 9 L of chylous fluid with elevated triglyceride levels. Conservative management, including a medium-chain triglyceride (MCT) diet and somatostatin analogues, failed to achieve resolution. Lymphography subsequently identified multiple leak sites in the right groin; however, an attempt at percutaneous embolization was unsuccessful. Definitive surgical exploration was performed, during which the mesh was removed, revealing injured lymphatic vessels at the femoral ring. These vessels were ligated, and Tisseel® (Baxter) fibrin sealant was applied. The patient recovered uneventfully, with no recurrence at the three-year follow-up.

This case highlights that lymphatic malformations can mimic inguinal hernias and lead to severe iatrogenic complications if not considered in the differential diagnosis when clinical findings or imaging are ambiguous. When conservative measures fail, surgical intervention, specifically mesh removal and direct ligation, is essential. In this patient, the presence of the mesh likely perpetuated the lymphatic leak. This complex scenario required multidisciplinary management; ultimately, prevention relies on accurate preoperative diagnosis.

## Introduction

Chylous ascites is the accumulation of triglyceride-rich lymphatic fluid within the peritoneal cavity due to disruption or obstruction of lymphatic ducts [1]. This usually occurs due to trauma and rupture of the lymphatic vessels or increased peritoneal lymphatic pressure secondary to obstruction [2]. Spontaneous chyle leak is a rare occurrence, often associated with malignancy or cirrhosis [3]. Abdominal paracentesis is the most important test in diagnosing chylous ascites [2]. It presents as a milky liquid, dense and rich in triglycerides and chylomicrons. It is important to measure the levels of lactate dehydrogenase (LDH) in it, as well, as they also contribute to a diagnostic key.

The most striking clinical finding is abdominal distension, without associated pain. Symptoms such as diarrhoea, malnutrition, edema, or dyspnea may also occur [4]. Some patients develop significant malnutrition due to chylous nutritional loss associated with the injury to the lymphatic pathway. While more commonly associated with malignancy, trauma, or lymphatic malformations, the occurrence of chylous ascites following laparoscopic transabdominal preperitoneal (TAPP) hernia repair is exceedingly rare. 

Normally, the Canal of Nuck (processus vaginalis in females) obliterates within the first year of life [5]. Hydrocele of the Canal of Nuck constitutes a type of primary idiopathic hydrocele, which has been attributed to a defect of the secretory membranes resulting in an imbalance in secretion and absorption of fluids [5]. Principal causes of postoperative lymphatic leak are radical lymph node dissection of the iliac vessels and/or paraaortic lymphadenectomy. Some novel techniques to prevent lymphatic injury, such as subcutaneous injection of indocyanine green (ICG) in the dorsum of the foot and the thigh below the injury, have been reported. This allows for real-time fluorescence visualization and preservation of stained lymphatic ducts during dissection [6].

Hernia surgery exceptionally causes lymphatic leak; most commonly, the complication consists of seroma or haematoma contained within the mesh surroundings. This case underscores a challenging and persistent chyle leak resulting in massive ascites, nutritional deterioration, and prolonged hospitalization. The most important step in the management of chylous ascites is to optimize the patient's nutritional status. This includes the use of a high-protein, low-fat diet supplemented with medium - chain triglycerides. Medium - chain triglycerides are absorbed by the enterocytes and transported as free fatty acids and glycerol directly into the liver through the portal vein, sparing the lymphatic system [7]. 

Total parenteral nutrition (TPN) allows for digestive rest and a decrease in splanchnic flow and secondarily in chylous production [4]. TPN is the cornerstone in medical management, thus contributing to restoring nutritional status. 

We present the case of a 35-year-old female who underwent a TAPP right inguinal hernia repair at an outside institution. The procedure was complicated by chylous ascites due to iatrogenic injury of an inguinal lymphatic malformation that had been misdiagnosed as a hernia. Despite multiple reinterventions, she presented to our center with severe malnutrition and high-output chyle drainage. Definitive resolution was achieved through surgical re-exploration, mesh removal, direct lymphatic ligation, and the application of fibrin sealant (Tisseel®, Baxter).

Its uniqueness lies in its etiology, recurrence, and successful surgical outcome after exhaustive conservative management.

## Case presentation

A 35-year-old woman, previously healthy, underwent a laparoscopic right-sided TAPP inguinal hernia repair with mesh at an outside institution in May 2022. During the procedure, a cystic lesion suggestive of a Nuck’s duct cyst was incidentally identified and drained. A self-fixating Progrip® mesh was placed in the preperitoneal space. Twenty-two days postoperatively, the patient presented with progressive abdominal distension and discomfort. An abdominal CT scan revealed large-volume ascites. She underwent laparoscopic exploration, which confirmed the presence of chylous ascites. Due to active leakage, the procedure was converted to open surgery with clip placement over presumed lymphatic leaks, followed by insertion of an abdominal drain. 

**Figure 1:**
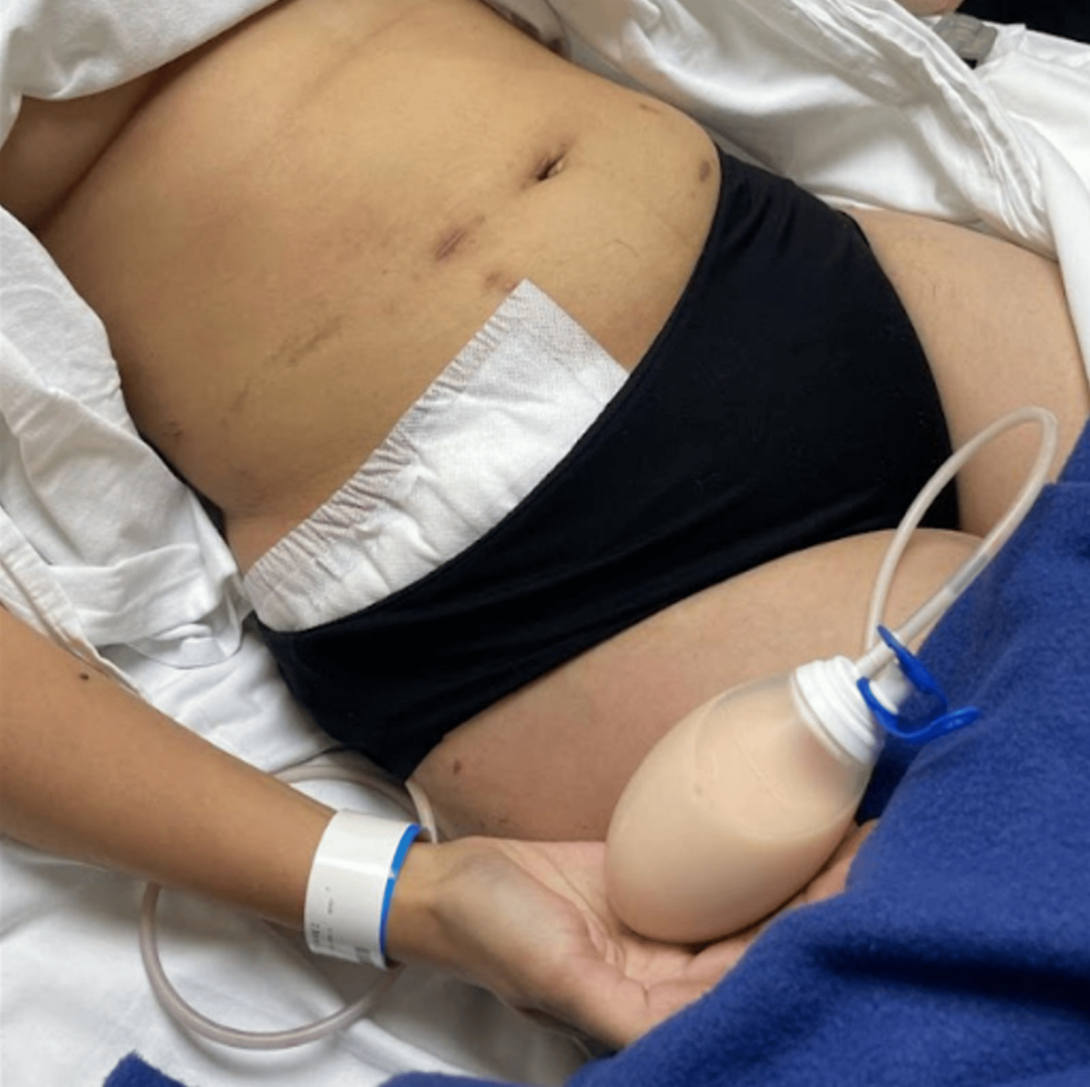
Patient upon referral to our center with her drainage with chylous fluid.

Although the ascites initially resolved, it recurred two months later, prompting a second laparoscopic reintervention where a closed-suction drain was placed in the right inguinal region, with persistent drainage of approximately one liter per day of chylous fluid (Figure 1).

**Figure 2:**
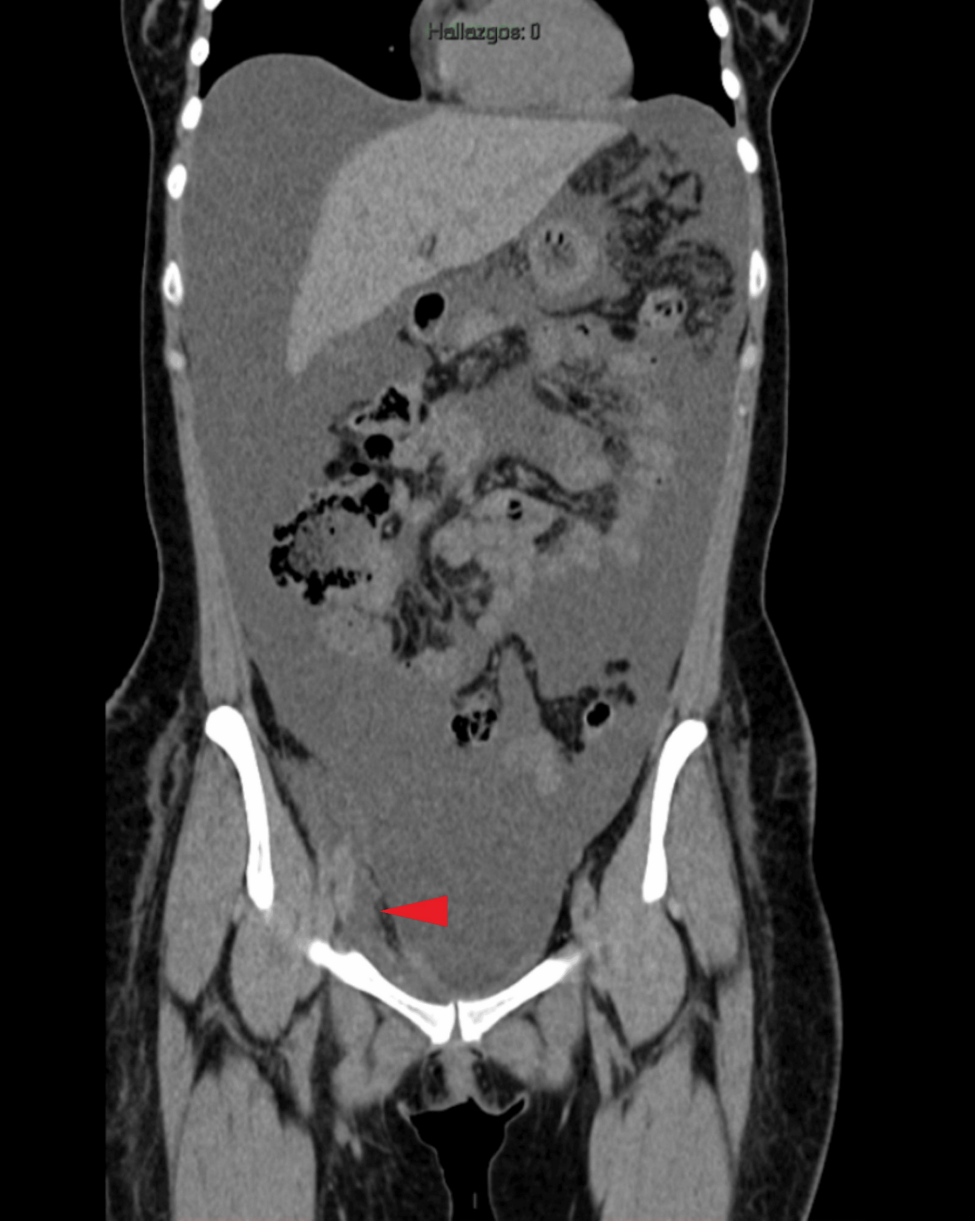
CT scan showing peritoneal cavity with massive ascites and the fluid collection in the right groin (red arrowhead). CT: Computed tomography

The patient then underwent two sessions of sclerotherapy with polidocanol (Aethoxysklerol®) without clinical improvement. Six months after the index procedure, the patient was referred to our center for further evaluation and management. Upon arrival, she was markedly malnourished, weighing 50 kg (BMI ≈ 18.4 kg/m²), with hypoalbuminemia (3.03 g/dL) and significant muscle wasting. CT imaging demonstrated massive free peritoneal fluid and a 6.7 × 3.5 cm fluid-filled lesion adjacent to the right iliac vessels, suggestive of a lymphocele with subcutaneous extension (Figure 2). Paracentesis yielded nine liters of chylous fluid with elevated triglycerides.

**Figure 3:**
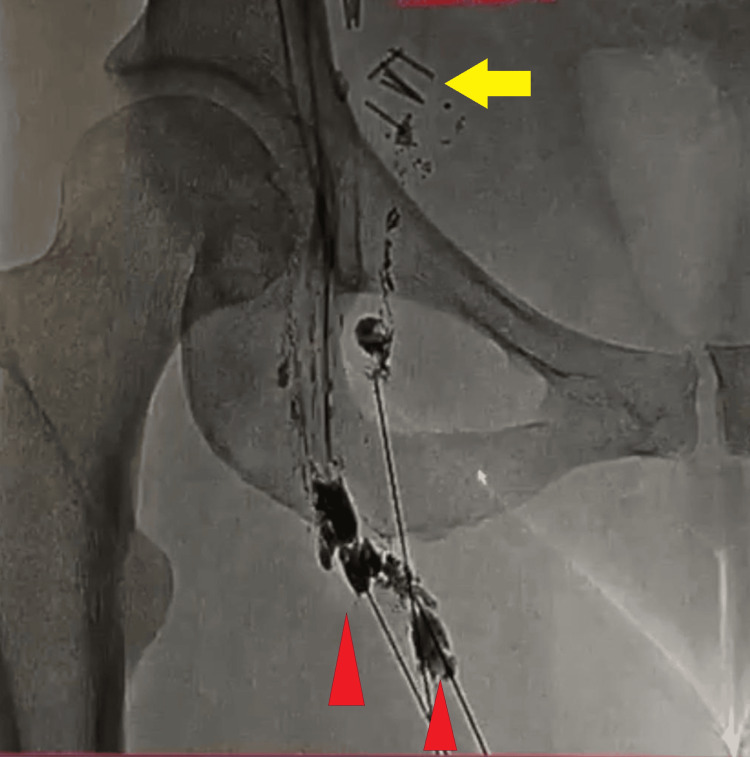
Lymphography showcasing multiple leakage sites on the right groin (red arrowheads). Note the presence of clips from the previous procedures (yellow arrow).

Lymphography confirmed multiple leak sites in the right groin (Figure 3), and a subsequent attempt at percutaneous embolization with cyanoacrylate was unsuccessful.

**Figure 4:**
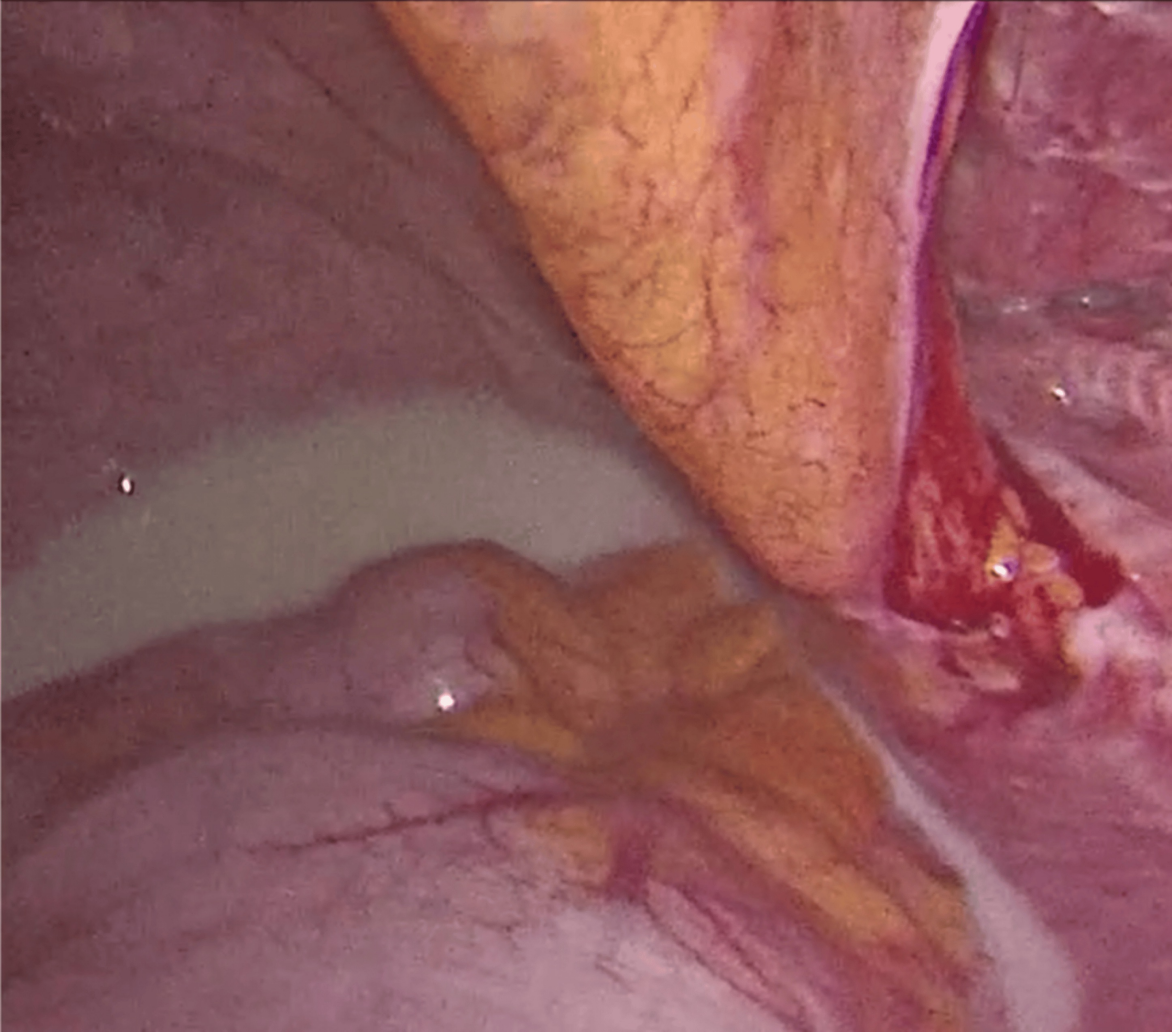
Chylous ascites vissible in pelvis and deperitonealized inguinal region with folded mesh and chylous leaking underneath.

**Figure 5:**
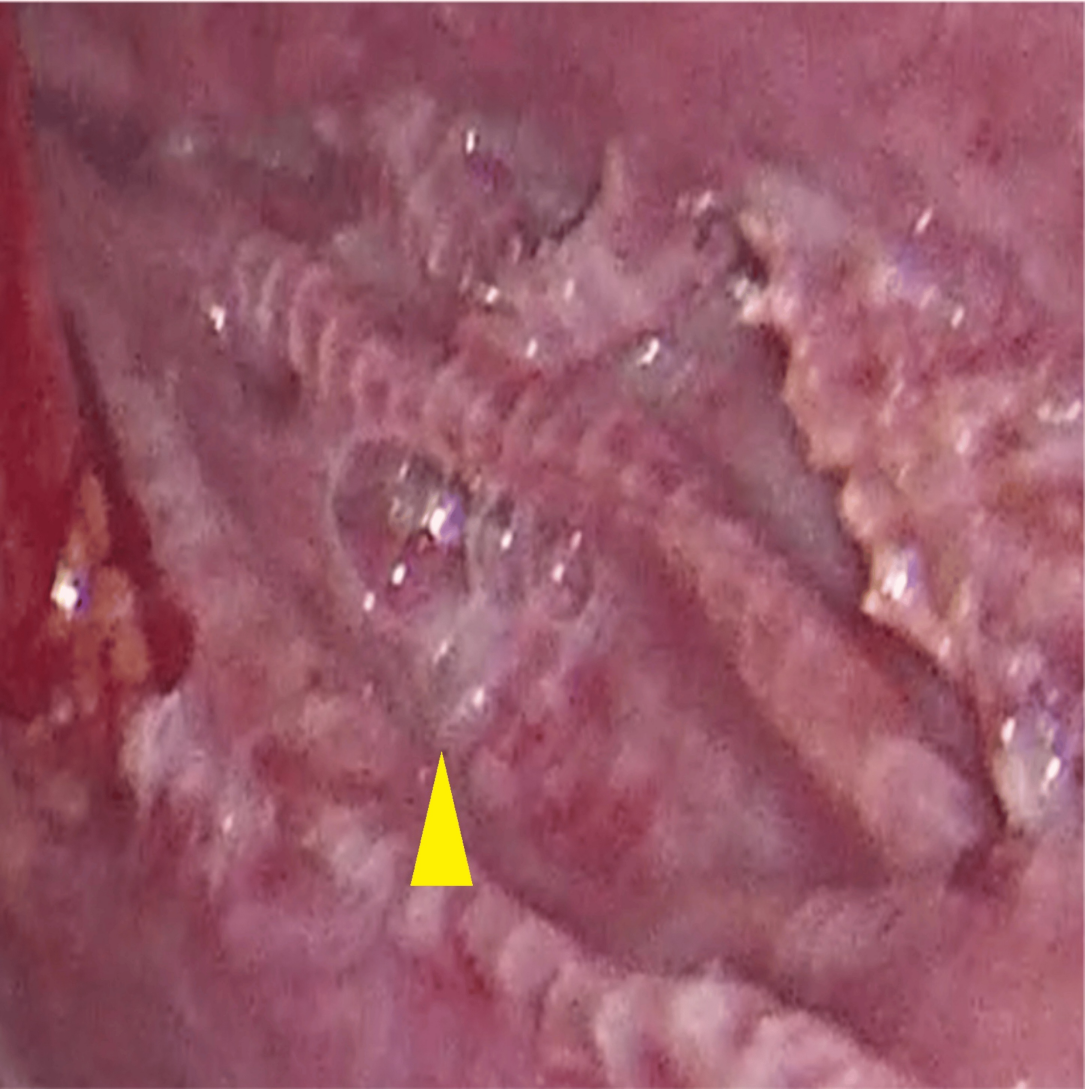
Leakage site evident upon palpation of the right groin.

A laparoscopy was performed, aiming to remove the mesh, revealing abundant chylous fluid, a folded and deperitonealized mesh, and active leakage of chyle upon palpation of the right iliac fossa (Figures 4, 5).

The leak appeared to originate from a disrupted retroperitoneal lymphatic malformation near the right iliac vein (Video 1).

**Video 1:** Laparoscopic removal of the mesh prior conversion to open surgery.

**Figure 6:**
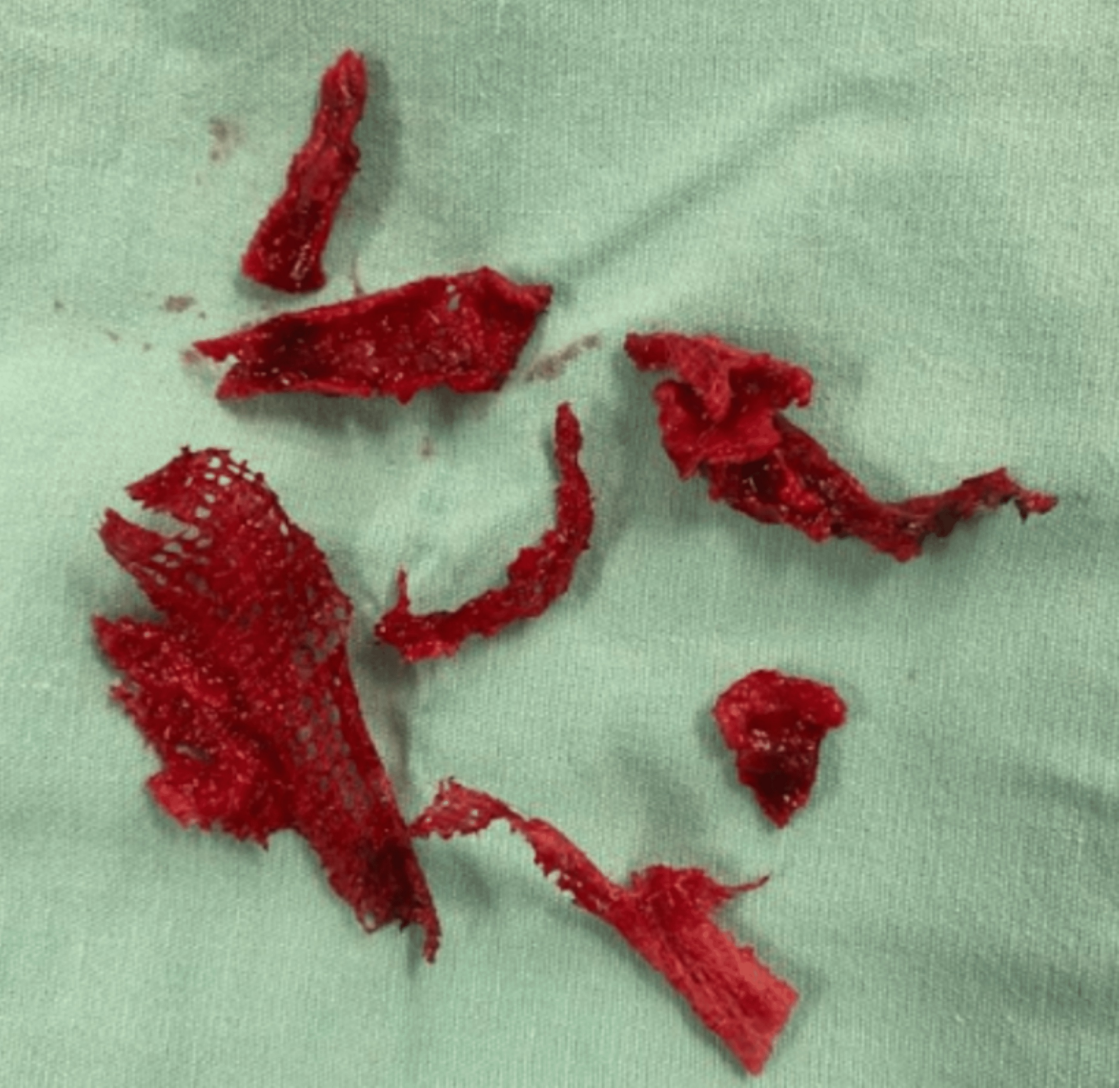
Mesh removed in pieces due to partial integration.

The procedure was converted to midline infraumbilical laparotomy, with the removal of the mesh, which was partially integrated and had to be extracted in fragments (Figure 6).

**Figure 7:**
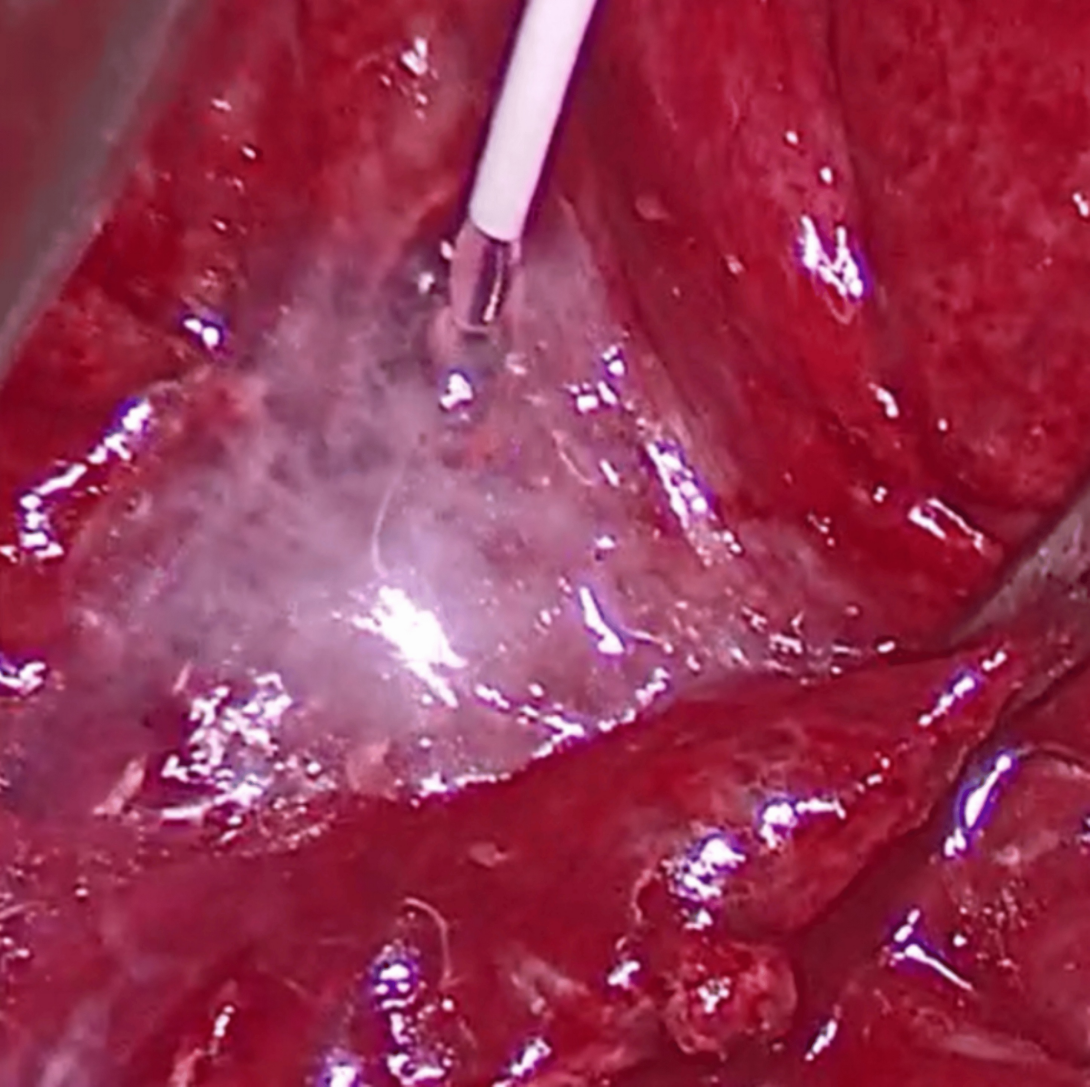
Application of Tisseel®, Baxter fibrin sealant on the leak.

The identified lymphatic leak was directly ligated using 4-0 monofilament sutures, and 14 mL of Tisseel®, Baxter fibrin sealant was applied (Figure 7).

Peritonization of the area was completed, and two 19 Fr Blake® drains were left in situ. Intraoperative blood loss was minimal (~100 mL), and a triple-lumen central venous catheter was placed for total parenteral nutrition (TPN).

**Figure 8:**
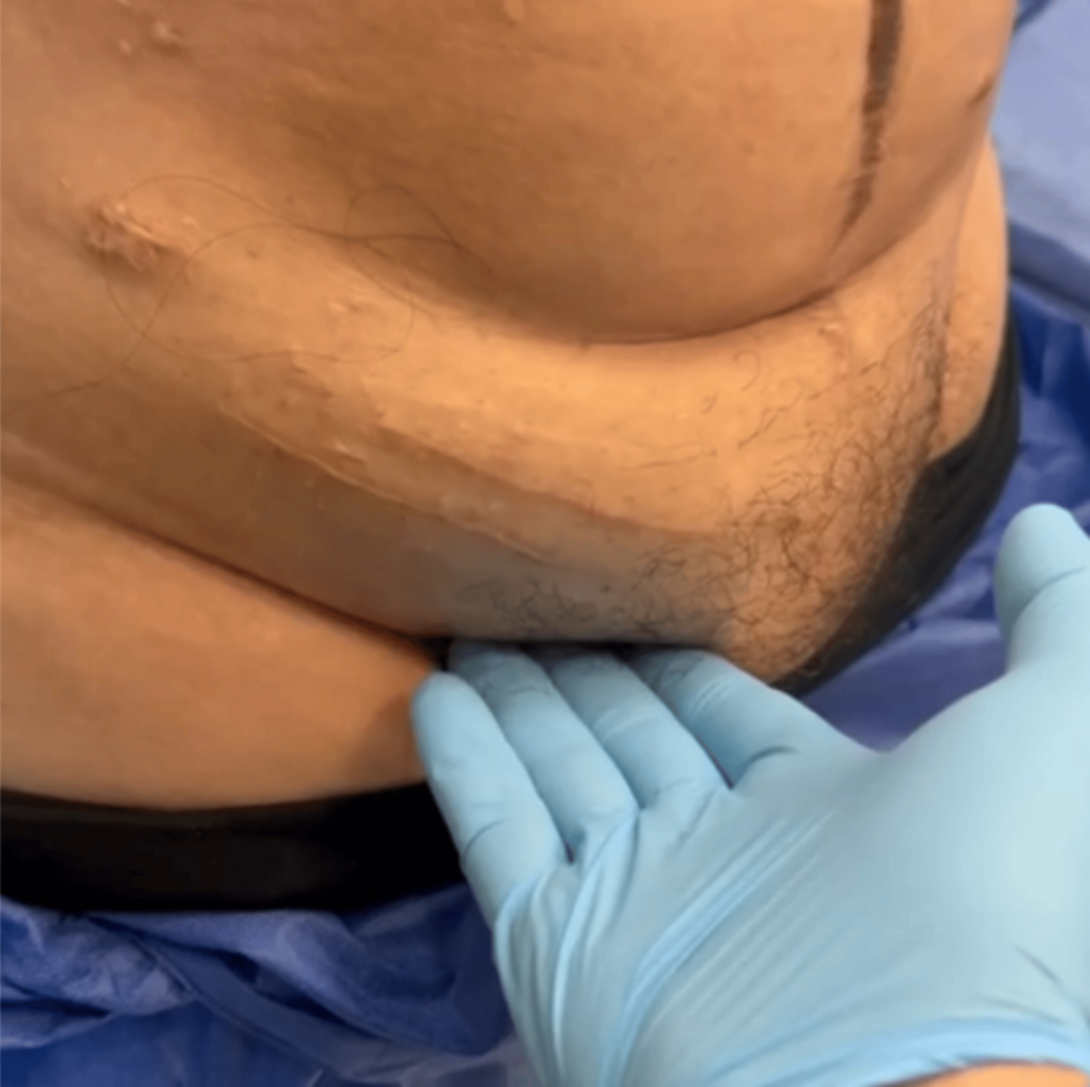
Persistent right inguinal bulge three years after surgery.

**Figure 9:**
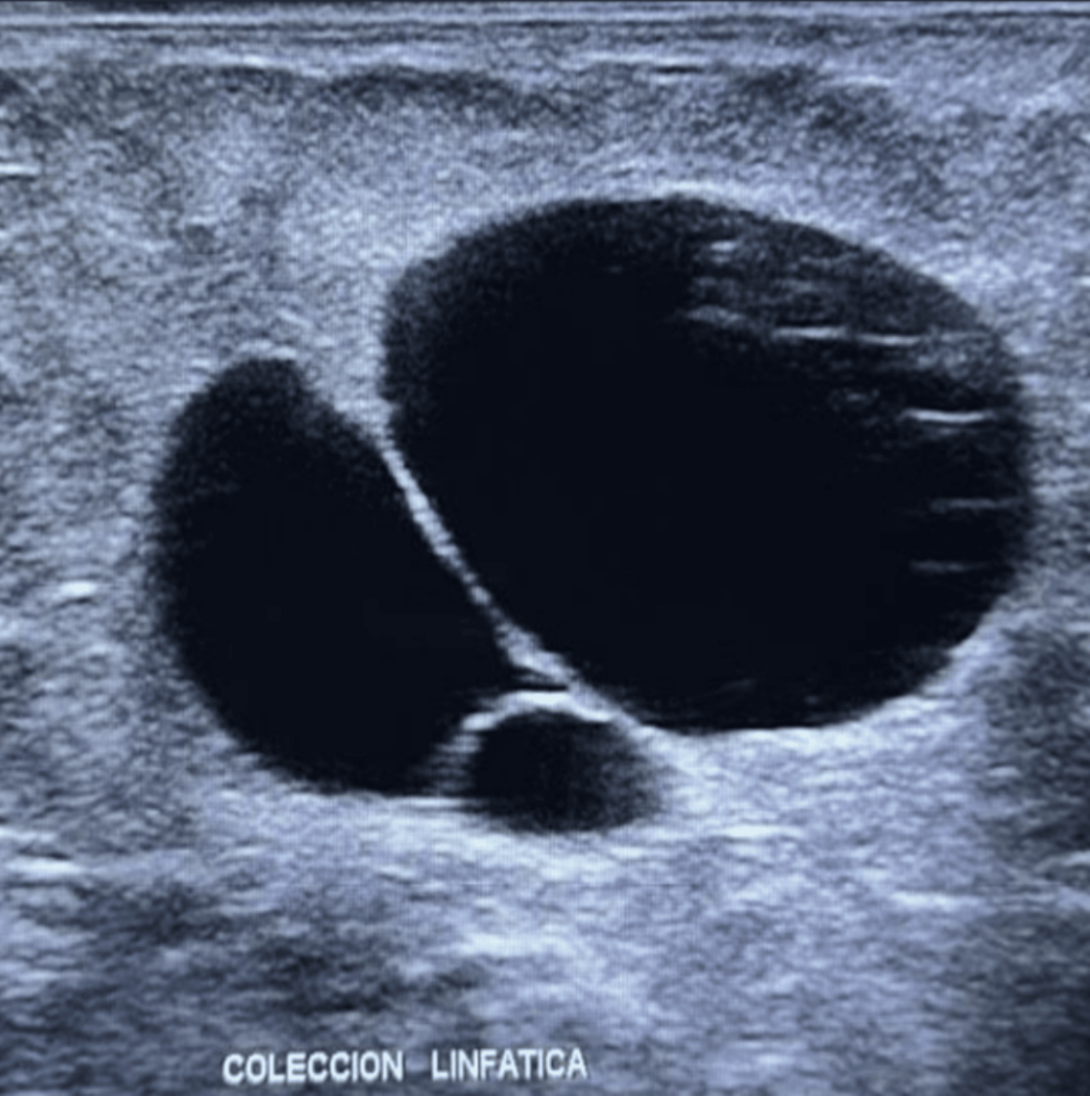
Ultrasound showing remnant cystic collection in the right groin three years after surgery.

The patient remained hospitalized for 22 days, receiving TPN, octreotide, and progressive introduction of medium-chain triglyceride (MCT)-based diet. Drain output steadily decreased and was serous by day 12 and 15, when the drains were removed. The patient was discharged with a stable nutritional status and follow-up arrangements. At the three-year follow-up, the patient is completely asymptomatic, with no recurrence of ascites. Her nutritional markers are normal, and she has regained healthy body weight (BMI ≈ 21.4). A small, non-tender right inguinal bulge persists, likely corresponding to a congenital lymphatic sinus, initially mistaken for an inguinal hernia. A recent ultrasound confirmed a stable, non-vascularized cystic remnant with no evidence of recurrence (Figures 8, 9).

## Discussion

This case represents a unique cause of chylous ascites following TAPP hernia repair, and no other case has previously been reported to the best of our knowledge, in PubMed, BMJ, LILACS and Cochrane Library databases.

The increasing adoption of the TAPP technique worldwide has created a unique educational paradigm in our setting. While public hospitals in Mexico still rely on open repairs due to budget constraints, our private center’s high volume of laparoscopic cases has inverted the traditional training sequence. Consequently, residents are developing advanced laparoscopic skills before gaining significant experience in open surgery, reflecting a 'reverse' learning curve driven by resource availability.

Other causes of chylous ascites have been reported, such as pelvic and/or aortic lymphadenectomy, open or laparoscopic nephrectomy, pancreatitis with extensive necrosis, lymphatic malignancies and congenital causes. However, lymphatic inguinal malformations are rare in the adult population, especially unilateral. Inguinal hernia on the other side is a frequent cause of surgery, with 20 million groin hernia repairs being performed annually worldwide [8], which explains why the lymphatic malformation was initially mistaken for a right-sided inguinal hernia in this patient.

Chylous ascites are singularly rare after laparoscopic inguinal hernia repair and even more exceptional when presenting as recurrent high-output ascites. Its management requires a tailored approach that combines radiologic evaluation, nutritional support, and, when necessary, surgical resolution. 

The present case underscores the necessity of considering congenital lymphatic anomalies in the differential diagnosis of inguinal hernia. Although clinical examination may be adequate to justify surgery, imaging modalities such as inguinal ultrasound and CT scans are valuable adjuncts for diagnostic confirmation, particularly in excluding a true hernia when no fascial defect is identified. Conservative management, comprising bowel rest (NPO) and octreotide administration, is typically effective; however, the optimal duration of treatment before declaring medical management failure remains poorly defined. 

Zi Qin Ng and Bulang He proposed a classification system and therapeutic strategy for chyle leak after laparoscopic living-donor nephrectomy after studying 178 laparoscopic living-donor nephrectomies between 2005 and 2016. They classified chyle leak in three grades according to daily output: mild for less than 300 ml/day, moderate between 300 and 800 ml/day and severe chyle leak if >800 ml/day is quantified from the drainage. For mild chyle leak, the management they established was “nil per os” (bowel rest) for two weeks and subcutaneous octreotide at 100 μg three times/day. Moderate leak is treated with the same regime adding 100 to 200 μg of octreotide three times/day and protein supplementation with gradual introduction to regular diet afterwards. Finally, for severe chyle leak, two weeks of medical management, like in moderate grade, adding close surveillance, generally, surgical exploration is needed to avoid severe malnutrition and immune suppression. Another recommendation is to facilitate chyle leak identification by giving a high-fat diet to the patient prior to surgery for better appraisal [9].

A systematic review by Weniger M and D'Haese JG, assessing chylous ascites after major abdominal surgeries, ascertained that this surgical complication was more common after gastrectomy with D3 lymph node dissection (11.7%) and pancreatic surgery (1.0% to 11%) [10], but no similar cases were reported in the context of inguinal hernia repair. Further studies are needed to standardize chyle leak management regardless of the primary surgery which caused the leak. The current consensus is based primarily on the classification of the International Study Group on Pancreatic Surgery due to its non-negligible prevalence in pancreatic surgery [11].

In this particular case, we hypothesize that the presence of the mesh caused a persistent pro-inflammatory environment around the lymphatic ducts, preventing them from closing, although medical management was correctly indicated [9-10]. Therefore, mesh removal, ligation of the leak sites, and Tisseel®, Baxter fibrin sealant application, remained the cornerstone of the surgical management. As of the moment of this publication (three years after discharge), the patient's follow-up has not shown any relapse, and her nutritional status was reestablished.

## Conclusions

Chylous ascites after TAPP mesh repair is exceedingly rare. This case highlights the importance of an accurate hernia diagnosis and further radiological imaging when clinical or ultrasound findings are ambiguous. These patients require multidisciplinary management, including nutritional support. In this instance, the primary misdiagnosis of a lymphatic malformation led to unnecessary surgery and subsequent duct injury, resulting in chylous ascites. Surgical removal of the mesh, direct ligation of chyle leaks, and Tisseel®, Baxter fibrin sealant, resulted in the definitive resolution after long-lasting conservative management failed. Long-term follow-up confirms the durability and effectiveness of the intervention. Determining the right timing for surgical exploration might have mitigated the nutritional status of this patient. Early surgical exploration with mesh removal, direct ligation and Tisseel®, Baxter fibrin sealant remains the mainstay of surgical resolution.
